# Maternal-fetal interface organoids: a new era in pregnancy research

**DOI:** 10.3389/fbioe.2026.1842889

**Published:** 2026-06-09

**Authors:** Jingran Gao, Hangyu Sun, Shuqi Yang, Xiaoying Yao

**Affiliations:** Obstetrics and Gynecology Hospital of Fudan University, Shanghai Key Lab of Reproduction and Development, Shanghai Key Lab of Female Reproductive Endocrine Related Diseases, Shanghai, China

**Keywords:** maternal-fetal interface, organoids, placenta, pregnancy-related diseases, reproductive medicine

## Abstract

The maternal-fetal interface is the core site of pregnancy occurrence and maintenance. Its complex physiological and pathological processes have long been concentrated in two-dimensional cell models and animal models. Organoids represent a novel *in vitro* three-dimensional model that offers a powerful platform for elucidating the complexities of human pregnancy, investigating pregnancy-related diseases, and developing novel treatment strategies. Organoids primarily employ pluripotent stem cells or tissue-derived progenitor cells to generate miniature “organs” that emulate the configuration of placental villi and/or endometrium under the influence of specific factors. The development of organoids has undergone substantial advancement. In the domain of reproductive medicine, maternal-fetal interface organoids have emerged as a versatile tool for simulating embryo implantation, elucidating maternal-fetal communication mechanisms, and facilitating etiological investigation and disease modeling of significant pregnancy complications, including recurrent miscarriage and preeclampsia. Furthermore, they can play an instrumental role in the realm of drug screening and research on vertical transmission of pathogens. The advancement of this field is contingent upon the synergistic integration of organoid culture technology and research methodologies, including imaging and multi-omics analysis. However, the field of maternal-fetal interface organoids is still in its nascent stages and faces many challenges. These include limitations in cell type completeness, tissue complexity, and functional maturity of existing models. In addition, there are standardization, reproducibility, and related ethical issues of culture. In the future, the development of this field will be driven by several key directions. These include the construction of additional physiological models, the integration of the immune and vascular systems, the promotion of standardization and clinical translation, and the establishment of clear ethical guidelines. These developments are expected to ultimately lead to breakthroughs in unlocking the early secrets of life’s origins and preventing reproductive disorders.

## 1 Introduction

**FIGURE 1:**
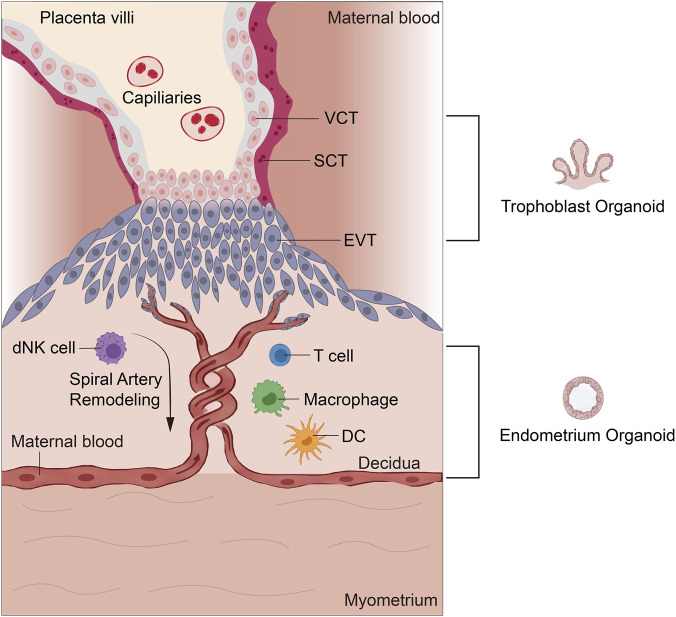
Composition of the maternal-fetal interface.

The maternal-fetal interface is a key structure for the establishment and maintenance of pregnancy, forming a highly complex microenvironment. It is composed of villous cytotrophoblasts (VCT), syncytiotrophoblasts (SCT), extravillous trophoblasts (EVT), decidual stromal cells, endothelial cells, and various immune cell populations-including decidual natural killer cells (dNK), macrophages, dendritic cells (DC), T cells, and other minor immune subsets-together with the cytokines they secrete (Figure 1). It has been demonstrated that this interface plays a multifaceted role in various physiological processes, including embryo attachment and implantation, placental development, nutrient exchange, gas exchange, and immune tolerance. Dysfunction of the maternal-fetal interface has been demonstrated to be closely related to various reproductive diseases, including infertility, early pregnancy failure, preeclampsia, and intrauterine growth restriction. Consequently, conducting in-depth research on the biological characteristics and pathological changes of the maternal-fetal interface is imperative to elucidate the mechanisms of pregnancy-related diseases and identify novel diagnostic and treatment strategies.

Research on the maternal-fetal interface has long been limited by models. Although traditional animal models play an important role in the study of embryo implantation and placental development (Ma et al., 2023). It should be noted that mouse uterine structure and embryonic orientation of rodents differ significantly from those of humans. Rodent models exhibit significant physiological differences in cycle length, with the mouse estrous cycle being 4–5 days, while the human menstrual cycle is 28 ± 7 days. There are also notable differences in menstruation. Giant cells in the mouse sinus trophoblast of the placenta have been shown to be invasive and to function in a manner analogous to human extravillous trophoblast cells. However, the invasive capacity of these cells is significantly less than that of equivalent numbers of human extravillous trophoblast cells (Ma et al., 2023). The ability of animal models to fully replicate the distinctive implantation patterns and placental structures observed in humans remains limited. *In vitro* cell culture models generally employ cell lines, including choriocarcinoma cell lines BeWo and JEG-3, and the extrachorionic trophoblast cell line HTR8. These cell lines are typically cultivated in two-dimensional conditions, which limits their ability to accurately replicate the three-dimensional structure and physiological dynamics of the maternal-fetal interface (Table 1) (Fatehullah et al., 2016). Consequently, there is an urgent need for an innovative model that can simulate the human maternal-fetal interface to reveal pregnancy-related mechanisms that more closely resemble *in vivo* conditions.

**TABLE 1:** Comparison of organoid, 2D cell culture, and animal model.

Model	Advantages	Limitations	Applicable research fields
Organoid	- Recapitulate 3D tissue architecture and heterogeneity of native organs - Derived from patient/human cells, preserving genetic background and reducing species differences - Amenable to long-term culture, cryopreservation, and biobanking - Support high-throughput drug screening and genetic manipulation (e.g., CRISPR/Cas9) - Generally faster and less costly to establish than animal models	- Lack integrated vasculature and immune components, limiting organ-scale maturation - Dependence on exogenous matrix (e.g., Matrigel) with variable composition - May not fully capture whole-body physiology or inter-organ interactions	- Disease modeling - Drug discovery and toxicology testing (efficacy/safety screening) - Stem cell and developmental biology research - Precision/personalized medicine (patient-specific organoids)
2D cell culture	- Simple, well-established, and low-cost; easy to maintain and expand - Suitable for high-throughput assays, genetic engineering, and mechanistic studies	- Lack 3D structure and complex microenvironment (cells grow flat, losing normal polarity) - Cannot capture multicellular tissue architecture or physiological gradients - Often represent a single cell type and do not reflect *in vivo* heterogeneity	- Fundamental cell and molecular biology (signal transduction, genetics) - Initial drug screening and toxicity assays - Viability, metabolic, and mechanistic studies in pharmacology and toxicology
Animal model	- Provide complete *in vivo* physiology (multi-organ, immune, endocrine systems) - Enable assessment of systemic drug effects (pharmacokinetics, efficacy, toxicity)	- Species and genetic differences often limit relevance to human biology - High cost, ethical concerns, and long experimental timelines - Not all human diseases or drug responses can be replicated in animals	- *In vivo* pharmacology and toxicology - Disease models (cancer, metabolic, neurological, infectious, etc.) for preclinical research - Physiology and developmental studies; tissue engineering/regenerative medicine testing

## 2 Organoid research

Organoids are three-dimensional culture systems derived from stem cells or tissue-specific cells (Lancaster and Knoblich, 2014). These organoids have the capacity to self-organize *in vitro* to form structures and functions similar to the original organs. In 2009, Hans Clevers’ team at the Hubrecht Institute in the Netherlands achieved a significant milestone in stem cell research by successfully cultivating adult stem cells *in vitro* to form crypts and villi structures of the small intestine, thereby officially opening up this field of research (Sato et al., 2009). The advent of organoids has engendered a novel opportunity to address the aforementioned dilemmas. In comparison with two-dimensional cell culture, organoids possess the capacity to preserve the proliferative and differentiative capabilities of cells over an extended duration. Moreover, they offer a more accurate representation of *in vivo* cellular diversity and spatial organization. The present focus of organoid technology development is on enhancing biomimicry, that is, achieving enhanced simulation of the morphology, structure, and physiological function of tissues and organs in natural and disease states, narrowing the gap with real tissues and organs, and on this basis, exploring the realization of correlation between organoids and establishing multi-organ combined systems. In recent years, stem cell biology and three-dimensional culture technology have advanced to a point where organoids can be utilized in research related to a variety of organs, including the intestine, liver, brain, and heart (Huang B. et al., 2025; Wang et al., 2025c). The gut is the first organ type to be simulated by organoid technology. Following the establishment of the initial small intestinal organoid, researchers have since elucidated the culture conditions for various cells in the small intestine, including intestinal epithelial cells, goblet cells, and enteroendocrine cells (Basak et al., 2017). Brain organoids have emerged as a current research focus. Researchers have achieved the development and maturation of human brain organoids in rat brains and established connections with synapses of rat neurons, thereby enabling control of rat behavior (Revah et al., 2022). The first *in vitro* self-organizing heart organoid was successfully cultured using human pluripotent stem cells. It has been observed to spontaneously form cavities and beat autonomously, and it has also been demonstrated to autonomously mobilize cardiac fibroblasts to repair damage (Hofbauer et al., 2021), representing a breakthrough in heart organoids.

Moreover, the advent of trophoblast organoids, endometrial organoids, and various reproductive tract organoids has engendered novel breakthroughs in the domain of reproductive medicine (Wang et al., 2025a). Researchers have successfully established endometrial and trophoblast organoids and, to a certain extent, reproduced key physiological processes at the maternal-fetal interface, such as the cyclical changes of the endometrium, the formation of the embryo implantation window, and the differentiation and invasion of the trophoblast (Turco et al., 2018; Ciprietti et al., 2025). These organoids have engendered entirely new research pathways for exploring embryo implantation mechanisms, studying pregnancy complications, screening potential drugs, and developing personalized treatment strategies. Despite the rapid advancements in organoid research, numerous limitations persist. These include the absence of immune cells and vascularized components in the models, and the inability to fully replicate the intricate maternal-fetal immune tolerance environment. Moreover, constrained sample acquisition, variable construction standards, and impediments to clinical translation further constrain their extensive implementation. Consequently, a comprehensive review of the most recent research advancements concerning maternal-fetal interface organoids, accompanied by a thorough examination of their potential applications and the prevailing challenges, is imperative to foster the advancement of this domain. The subsequent analysis will examine the current state of research and its progress in the field of maternal-fetal interface organoids. This analysis will also discuss the potential applications of these organoids, with the aim of providing a reference point and inspiration for relevant researchers.

## 3 Organoid studies at the maternal-fetal interface

### 3.1 Definition

Maternal-fetal interface organoids can recapitulate certain cellular components and selected functions of maternal-fetal interface tissues, although they do not fully capture the complexity of *in vivo* conditions. The present study explores the proliferation and differentiation process of trophoblast cells under physiological conditions. These organoids are conventionally referred to as “trophoblast organoids” or “placental villous organoids” (Turco et al., 2018; Huang et al., 2023). In a broader sense, maternal-fetal interface organoids, in addition to trophoblast cell organoids, should also include decidualizable endometrial organoids, or other organoid culture systems containing both fetal and maternal components (Bondarenko and Turco, 2025). The construction of maternal-fetal interface organoids is mainly based on 2 cell sources: human trophoblast stem cells (hTSCs) and human pluripotent stem cells (hPSCs). The former are predominantly derived from placental tissue and can be directly cultured and differentiated into SCT and EVT in a three-dimensional matrix using the tissue block method or enzymatic digestion method (Okae et al., 2018). The latter include human embryonic stem cells (hESC) and human induced pluripotent stem cells (hiPSC), which require specific differentiation induction pathways to induce hPSC to derive hTSC(Castel and David, 2022; Jang et al., 2022).

### 3.2 Construction of maternal-fetal interface organoids

#### 3.2.1 Construction of trophoblast organoids

Trophoblast organoids have been observed to form villi-like structures in three-dimensional culture systems. These organoids possess the capacity to differentiate into various types of trophoblast cells, including cytotrophoblast (CTB), syncytiotrophoblast (STB), and extravillous trophoblast (EVT). In this manner, they reproduce critical aspects of placental development *in vitro*, thereby providing an ideal model for exploring the mechanisms of placental diseases.

A significant number of researchers have achieved groundbreaking advancements in the field of optimizing trophoblast organoid culture media. A multitude of studies have substantiated the pivotal functions of Wnt and EGF pathway activation, along with the suppression of TGF-β and ROCK pathways, in the proliferation and differentiation of trophoblast cells (Okae et al., 2018; Meinhardt and Haider, 2025). In this regard, EGF and TGF-β inhibitors, such as A83-01, ROCK inhibitor Y27632, Wnt activator CHIR99021, and R-spondin, have emerged as pivotal constituents of organoid culture media. Haider et al. found that trophoblast organoids maintained their condition well in media supplemented with Noggin (Haider et al., 2018). Sheridan et al. found that they could be maintained for multiple generations in media containing FGF2 (Sheridan et al., 2020).

In the context of specific cultural conditions, trophoblast organoids have been observed to exhibit differentiation and phenotypic characteristics reminiscent of trophoblast cells. Turco et al. constructed trophoblast organoids in which stem cells possessed the ability to self-renew and differentiate into syncytiotrophoblast and extravillous trophoblast. The researchers employed human leukocyte antigen (HLA) typing to ascertain the origin of the organoids, and they verified their origin based on trophoblast cell specificity criteria. Methylome analysis revealed that trophoblast organoids exhibited a high degree of similarity to normal early pregnancy placentas (Turco et al., 2018). Shannon et al. employed a combination of single-cell sequencing and metabolomics to not only confirm the consistency between the organoids and the villi of the original pregnancy tissue but also to further elucidate the differentiation pathway of trophoblast cells, providing reliable data for the study of trophoblast cell developmental disorders (Shannon et al., 2024). Yang developed a mildly oscillating suspension culture system that enabled trophoblast organoids to reproduce the polarity of trophoblast cells under *in vivo* physiological conditions. The determination was made through capacitance measurements that the outermost surface of the cultured organoids was a syncytiotrophoblast, with small syncytiotrophoblasts and mononuclear cells on its inner side (Yang et al., 2023). Building upon this, Hori’s organoids, cultured using a columnar culture device, also exhibited physiological polarity and reproduced the maternal-fetal interface barrier function. This model can be used to deduce the permeability coefficient of compounds at the maternal-fetal interface, allowing for the assessment of compound transfer and toxicity (Hori et al., 2024). Studies have found that culturing hTSCs using collagen IV-coated polystyrene beads, where hTSCs attach to the surface of the microbeads for growth, and then embedding both in a matrix gel for proliferation and differentiation, can also reproduce the *in vivo* polarity of SCT and VCT cells (Zhou et al., 2025). To more realistically simulate blood flow at the maternal-fetal interface, researchers are also exploring the integration of microfluidics to culture human induced pluripotent stem cells in a perfusion-type 3D culture fluid control device, promoting the differentiation of hiPSCs into trophoblast cells to construct trophoblast organoids (Deng et al., 2022).

#### 3.2.2 Construction of endometrial organoids

The endometrium, as a pivotal interface for interaction between the mother and the developing embryo, facilitates embryo implantation, development, and eventual successful delivery. Turco et al. developed the first human endometrial organoid model and found that long-term genetically stable organoids could be established from both non-pregnant endometrium and decidua. This organoid model demonstrated a response to sex hormones and exhibited molecular characteristics of glands in a living organism (Turco et al., 2017). The use of endometrial organoids has emerged as a valuable tool for studying the pathophysiology of the human endometrium. These models have been shown to overcome certain limitations, such as the lack of biological three-dimensional conditions and physiological phenotypes (Jabri et al., 2025). In the context of hormone-free culture conditions, the predominant cellular state of endometrial organoids is characterized by proliferation, accompanied by the expression of estrogen receptors, thereby underscoring their responsiveness to estrogen (Fitzgerald et al., 2019; Garcia-Alonso et al., 2021). The stimulation of organoids with estrogen has been demonstrated to induce progesterone receptor expression. When organoids are exposed to progesterone, cAMP signaling is activated, resulting in the production of a decidualized endometrial phenotype that is comparable to its *in vivo* state. This phenotype exhibits high expression of late-differentiation stage secretions, glands, and ciliated cells (Turco et al., 2017; Boretto et al., 2019). To more accurately mimic the multicellular structure and function of the endometrium, Rawlings et al. co-cultured epithelial organoids with endometrial organoids, forming glandular endometrial organoids containing matrix (Rawlings et al., 2024). Other studies have developed air-liquid interface endometrial assemblages, which have been shown to successfully preserve matrix, glands, and epithelial cells within endometrial epithelial organoids. These studies have also demonstrated the derivation of cleaving cilia (Tian et al., 2023). Researchers have utilized the interaction between the Wnt and Notch pathways to establish a lumen-glandular gradient in the endometrium. In the context of culturing endometrial organoids, the strategic downregulation of the Wnt pathway has been observed to yield a low proportion of ciliated cells and a high proportion of secretory cells. Conversely, the opposite effect is observed when the Notch pathway is targeted for downregulation (Garcia-Alonso et al., 2021).

### 3.3 Research progress of maternal-fetal interface organoids in reproductive medicine

**FIGURE 2:**
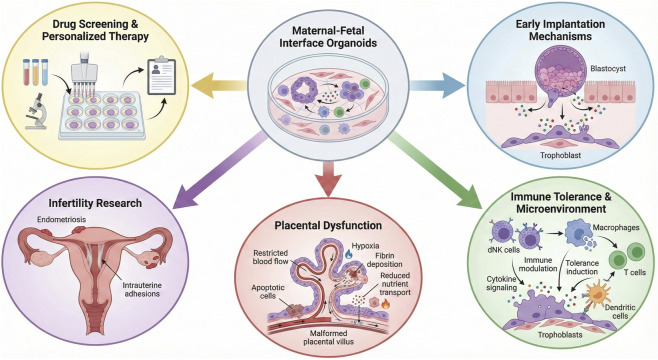
Applications of maternal-fetal interface organoids in reproductive research.

The development of maternal-fetal interface organoids offers a new way for research in the field of reproductive medicine (Figure 2).

#### 3.3.1 Maternal-fetal interface organoids and early pregnancy

The initial phase of pregnancy, often referred to as the “early pregnancy,” is characterized by a high degree of complexity and significance. This period represents a critical interaction between the developing embryo and the maternal environment. The blastocyst engages with the endometrium, penetrating it and inducing implantation. This process involves trophoblast cell adhesion, invasion, and decidualization of the endometrium. The success of pregnancy is determined by factors including endometrial receptivity, embryo quality, and the maternal microenvironment.

The endometrium, the site of embryo implantation and nutritional support, plays a crucial role in the initial phases of pregnancy. Existing endometrial organoids have been demonstrated to reproduce the functional changes of the proliferative and secretory phases under hormonal influence, thereby simulating the so-called “implantation window” (Turco et al., 2017; Ciprietti et al., 2025). However, suitable *in vitro* research models are lacking for early embryo implantation studies. The construction of an “open-faced endometrial layer” based on a closed endometrial organoid model has been demonstrated to expose the epithelial surface of the organoids, thereby facilitating the study of the embryo implantation process. Researchers have co-cultured endometrial organoids with trophoblasts derived from sheep intracytoplasmic sperm injection (ICSI) embryos, observing that the embryos can attach to the organoid surface and engage in molecular-level communication with it. The cyst-like structures generated by the trophoblast have been shown to further reveal the structural and developmental characteristics of the embryo implantation process (Saadeldin et al., 2025). Shibata et al. (2024) co-cultured endometrial organoids with human embryonic stem cells. The epithelial cells of the endometrial organoids were subsequently destroyed by syncytial cells, which then invaded and fused with endometrial stromal cells. Song et al. (2026) constructed a three-dimensional co-culture system of human embryo-endometrial organoids, realizing the development of human post-implantation embryos to day 14 post-fertilization under the support of endometrial organoids in an *in vitro* environment. In constructing embryo implantation models, some researchers have also started with blastocysts. The induction of “blastocyst”-like organoids from juvenile human pluripotent stem cells is achieved through the triple inhibition of the Hippo, TGF-β, and ERK pathways. The sequence and time of blastocyst development are critical factors in the generation of analogs of three lineages (trophoblast, ectoderm, and primitive endoderm), which endow embryonic cells with the capacity to attach directionally to hormone-stimulated endometrium, thereby simulating the implantation process (Kagawa et al., 2022). Li et al. (2026), Song et al. (2026) have also developed a 3D co-culture system for human blastoids or blastocysts with endometrial organoids, which can reflect the developmental process after implantation.

The success of a pregnancy is contingent upon the maintenance of immune balance between the mother and the fetus. Pregnancy, a distinct form of allogeneic graft, facilitates the expression of cell surface molecules and the production of soluble cytokines by trophoblastic cells derived from the embryo. These molecules and cytokines contribute to the regulation of maternally derived immune cells, thereby inducing specific immune tolerance to embryonic antigens. The maternal-fetal interface is comprised of a variety of immune cells, including decidual natural killer cells (dNK, CD56^+^CD16^−^), macrophages, T cells, and B cells. The collaborative actions of these cells are instrumental in preserving embryonic tolerance and maintaining local immune homeostasis. This is achieved through the secretion of factors and direct cell-to-cell contact. Organoid systems that have been traditionally developed have typically exhibited a paucity of immune components. For instance, the trophoblastic organoids and endometrial organoids that were initially constructed by Turco et al. did not contain immune cell components (Turco et al., 2017; Turco et al., 2018). This limitation results in a significant restriction to the scope of research on maternal-fetal immune regulation.

In recent years, researchers have introduced immune cells into organoid culture systems, attempting to construct organoid models containing immune cells. The present state of research on the introduction of immune components into organoid culture systems is focused on the field of oncology. Three primary methods have been identified. The initial approach involves the co-culturing of tumor organoids with immune cells isolated from autologous peripheral blood, followed by the acquisition of tumor-specific immune cells through cytokine stimulation (Dijkstra et al., 2018). The second method entails the matrix gel co-culture technique, wherein immune cells are introduced to the top or interior of the matrix gel once the organoids attain a specific size, thereby establishing an organoid-immune cell co-culture model. Dong et al. constructed a co-culture model of cervical cancer organoids and *in vitro* expanded T cells, analyzing the pathways and matrix that enhance the function of γδT cell effector molecules. This study contributes to the field by providing a framework for studying how to improve the efficacy of γδT cell immunotherapy for cervical cancer (Dong et al., 2023). In addition, there is an aero-liquid culture method that preserves the matrix and immune cells. The culture dish is subdivided into an inner and an outer part, and substances can be exchanged between these two regions through a biofilter membrane. The organoids are planted in the inner layer, and culture medium is added to the exterior, thereby enabling the organoids to obtain nutrients from the culture medium and to be exposed to air. This method preserves the *in-situ* microenvironment; however, the immune components will decay over time (Neal et al., 2018). During pregnancy, the maternal-fetal interface immune microenvironment is relatively autonomous from the peripheral immune environment. Preserving the *in-situ* immune microenvironment is more akin to the *in vivo* situation. Furthermore, the co-culturing of single-cell immune systems with organoids at the maternal-fetal interface has been demonstrated to have significant applications. The co-cultivation of a single immune cell facilitates direct observation of the function of a specific immune cell at the maternal-fetal interface. For instance, co-culturing uNK cells with trophoblast organoids can mirror their regulatory function in trophoblast invasion behavior; co-culturing macrophages with endometrial organoids can be utilized to examine the association between inflammatory responses and pregnancy failure.

#### 3.3.2 Maternal-fetal interface organoids and infertility

Endometriosis and intrauterine adhesions have been identified as significant contributors to infertility. Endometriosis is characterized by the presence of endometrial-like tissue outside the uterus, i.e., ectopic lesions. Organoids derived from ectopic endometriosis lesions have demonstrated superior capacity to mimic disease-specific characteristics. Changes in intracellular calcium ion levels have indicated that the functional expression of mechanosensitive PIEZO1 channels approaches that of primary endometrial epithelial cells (Hennes et al., 2019; Luddi et al., 2020). However, for voltage-gated channels, no increase in current density was detected after voltage-step stimulation in patch-clamp experiments, suggesting a potential absence of functional voltage-dependent calcium channels (Ciprietti et al., 2025). Consequently, although ectopic endometrial organoids also possess hormonal regulatory mechanisms, the *in vitro* culture microenvironment may be influenced by the absence of stromal cells.

Asherman’s syndrome (AS) is characterized by intrauterine adhesions and is typically caused by endometrial damage and abnormal repair resulting from curettage or infection. This condition leads to implantation failure, recurrent miscarriage, and placenta accreta. The development of patient-derived endometrial organoids for Asherman’s syndrome provides a model for studying this disease. In particular, they reflect changes observed in the endometrium *in vivo*, particularly in the Wnt and Notch signaling pathways (Santamaria et al., 2023). Transplanting healthy human endometrial organoids into a mouse model of AS with damaged endometrium not only reconstructed the structural integrity of the endometrial epithelium but also achieved functional repair of the endometrium through differentiation, culture, and secretion (Zhang et al., 2022).

#### 3.3.3 Maternal-fetal interface organoids and placenta diseases

Abnormal placental development is a significant cause of serious complications, including preeclampsia, placental abruption, and intrauterine growth restriction. Maternal-fetal interface dysfunction is closely related to various placental diseases, and organoids provide a new tool for modeling and exploring the mechanisms of these diseases. Trophoblast organoids have been demonstrated to reproduce villous structures and the differentiation process of trophoblast cells *in vitro*, thereby simulating early placental development. A body of research has demonstrated that trophoblast organoids resulting from hypoxia-induced preeclampsia manifest abnormalities, including inadequate trophoblast invasion, an elevated sFLT-1 (soluble fms-like tyrosine kinase 1)/PlGF (placental growth factor) ratio, and oxidative damage, providing direct evidence to support the hypothesis that this phenomenon can explain its pathogenesis (Huang S. et al., 2025). In addition, the authors of the study validated the pharmacological effects of aspirin using this model. Kreis et al. utilized trophoblast organoid models to investigate the role of the cell cycle regulator p21 in preeclampsia (Kreis et al., 2021), and Sheridan et al. noted that trophoblast organoids can be employed to study the development and function of trophoblast cells and to analyze placental products (Sheridan et al., 2020). In the future, organoids are expected to become an important platform for the early diagnosis and drug development of placental diseases.

The maternal-fetal interface fulfills a dual role during pregnancy, functioning as both a nutrient exchange point and an immune barrier. To a certain degree, it has the capacity to impede the immediate effect of pathogens, toxins, and environmental pollutants on the embryo. A substantial body of research has employed well-established trophoblast and decidual organoids to explore the mechanisms underlying vertical viral transmission [e.g., teratogenic viruses (Yang et al., 2022; Hatterschide et al., 2025)] and exposure to environmental pollutants [e.g., perfluorooctanoic acid (Li et al., 2025)]. These studies have examined the potential impact of these environmental factors on trophoblast function and, consequently, embryonic development. Subsequent research endeavors may elucidate the maternal-fetal interface’s mechanisms of obstruction and reaction to deleterious external factors, thereby providing a substantial theoretical foundation and experimental evidence for the prevention and intervention of adverse pregnancy outcomes, such as abnormal embryonic development and miscarriage caused by infections and environmental exposures during pregnancy.

### 3.4 Limitations and challenges of maternal-fetal interface organoids

While significant progress has been made in the construction and application of maternal-fetal interface organoids, this field remains in the exploratory and developmental stage, facing numerous challenges and limitations. The ensuing analysis will examine aspects such as model construction, standardization, and technology.

#### 3.4.1 Complexity and incompleteness of model construction

The maternal-fetal interface is a dynamic system formed by the interaction of various cell types, including endometrial epithelium, stromal cells, trophoblast cells, immune cells, and endothelial cells. Although current maternal-fetal interface organoid models have been shown to simulate certain structures and functions with a reasonable degree of accuracy, the vast majority of these models are derived from a single cell population (e.g., epithelium or trophoblast). A notable limitation of these models is the omission of key components such as immune cells and vascular endothelial cells, which is problematic given the importance of these components in simulating the microenvironment of the maternal-fetal interface. Research progress in organoid immunology has been previously delineated (Section 3.3.1, paragraphs 3 and 4), and researchers are exploring organoid vascularization. Zhao et al. (2023) employed an organoid co-culture system to investigate the interaction between trophoblast cells and endothelial cells, thereby revealing that a pro-angiogenic factor secreted by trophoblast cells, WNT2B, can promote vascularization within the villi. A study also reported an engineered placental organoid-based microphysiological system incorporating endothelial cells, which establishes a vascularized microenvironment more closely resembling *in vivo* conditions. The inclusion of vascular components was shown to support trophoblast viability and sustained growth, while also promoting differentiation and functional activity, as evidenced by enhanced secretion of immunomodulatory factors and increased activation of innate immune signaling pathways (Wang et al., 2025b). The maternal-fetal interface exhibits a highly spatiotemporally dynamic tissue structure *in vivo*, while the majority of existing organoids are spherical or gland-like, failing to accurately reproduce processes such as endometrial stromal remodeling and spiral artery remodeling.

#### 3.4.2 Technical standardization and repetitiveness issues

The technical pathways for maternal-fetal interface organoid research have yet to be fully standardized, with significant variations observed between laboratories. Firstly, there are variations in culture media. Different studies use different formulations of growth factors and cytokines, leading to differences in organoid phenotypes and functional performance, making direct comparisons of results difficult. According to reports, the composition of the culture medium used by laboratories such as Turco and Huang for culturing trophoblast organoids differs (Turco et al., 2018; Huang et al., 2023). Secondly, factors such as tissue origin, digestion methods, cell sorting techniques, and the selection of 3D matrix all influence the efficiency and stability of organoid construction. A number of studies have recently explored the potential of 3D-printed hydrogels as a 3D matrix for organoid construction (Richards et al., 2025). In comparison to matrix gels, the composition of hydrogels can be more precisely controlled, thereby reducing variability between batches. Finally, there are currently no universally accepted standards to assess the maturity, functional integrity, and similarity to *in vivo* tissues of maternal-fetal interface organoids, which limits cross-study comparisons and wider application.

#### 3.4.3 Barriers to clinical translation

Maternal-fetal interface organoids hold immense potential in disease models and drug screening, but their translation into clinical applications still faces multiple challenges. For instance, tissues from disparate patient sources exhibit variations in genetic background, hormone levels, and past medical histories, which may compromise the clinical applicability of the models. Moreover, the prevailing organoid culture methods are predominantly reliant on costly manual procedures, exhibiting a paucity of large-scale, automated production capabilities. This limitation restricts their extensive utilization in clinical testing and drug screening applications. Furthermore, the safety, accuracy, and reproducibility of organoids for predicting drug responses or personalizing treatments necessitate extensive clinical validation to ensure their reliability.

## 4 Prospect

As an innovative *in vitro* model, maternal-fetal interface organoids are gradually becoming a key tool in early pregnancy research and reproductive medicine. The subsequent discussion will explore potential future development directions in the following aspects:

The first point to be considered is the standardization and reproducibility of models. At present, substantial discrepancies exist among laboratories with regard to organoid sources, culture systems, and induction methods. This situation engenders a dearth of comparability among research results. In the future, internationally recognized operating procedures and quality control standards must be established to ensure the reproducibility and reliability of maternal-fetal interface organoids under different research contexts. Secondly, there is the issue of multi-omics integration and mechanism analysis. The integration of high-throughput technologies, including single-cell transcriptomics, spatial omics, proteomics, and metabolomics, will facilitate a more comprehensive elucidation of the molecular networks and regulatory mechanisms that underpin the maternal-fetal interface. The deep integration of organoids and multi-omics enables researchers to more accurately explore the molecular matrix of the maternal-fetal interface. Studies have shown that organoid models generally exhibit a high degree of transcriptomic similarity to their corresponding *in vivo* tissues, supporting their value as physiologically relevant *in vitro* systems (Huang et al., 2023). Multiple research groups have conducted single-cell RNA sequencing analysis to map the cellular atlas of the endometrium, and organoids can serve as one of the validation models for omics results (Ciprietti et al., 2025). Thirdly, the maternal-fetal interface organoid on-chip is examined. Organ-on-a-chip technology has been demonstrated to offer the advantage of simulating complex tissue microenvironments. The integration of organoid and organ-on-a-chip technologies facilitates the establishment of a dynamic fluid environment, thereby simulating hemodynamics and material exchange at the maternal-fetal interface. The development of a co-culture system comprising endometrial organoids and trophoblast organoids is essential for simulating the interaction between the embryo, trophoblast cells, and endometrium. Fourthly, enhancing ethical standards is imperative. It is imperative that the academic community, ethics committees, and regulatory authorities collaborate to establish a comprehensive ethical and legal framework that aligns with international standards. This collaborative effort is crucial for ensuring the compliance and transparency of research.

The advent of technological advancements has rendered organoids of the maternal-fetal interface, constructed from patient-derived cells, a promising avenue for personalized modeling. This strategy can be used to explore the etiologies of specific patients and provide personalized treatment plans for clinicians. The utilization of patient-derived organoids facilitates the simulation of individual differences and the screening of drugs, thereby facilitating the identification of the most suitable treatment for specific patients. For instance, in the context of endometriosis research, patient-derived organoids have demonstrated variable channel sensitivities (Hennes et al., 2019), thereby offering novel insights into the potential for targeted therapy. Conversely, organoids have the capacity to replicate maternal-fetal interface functions *in vitro*, thereby functioning as a platform for drug safety testing. For instance, researchers have employed trophoblast organoids to assess the impact of aspirin on the secretory function and invasive behavior of trophoblast cells, thereby substantiating clinical medication interventions for preeclampsia (Huang S. et al., 2025).

The potential applications of maternal-fetal interface organoids extend beyond the scope of disease mechanism and drug research; they also hold promise for use in regenerative medicine and clinical applications. Some women experience endometrial damage or even intrauterine adhesions due to surgical procedures or injuries, which can have a profound impact on fertility. A growing body of research has demonstrated the efficacy of mouse and human endometrial organoids as grafts in mouse models of post-traumatic endometrial regeneration disorders. These findings suggest that endometrial organoids have considerable potential as transplant materials for endometrial repair in the future (Zhang et al., 2022; Hwang et al., 2024). In the field of assisted reproduction, endometrial organoid models have been utilized to detect the hormonal response and receptivity of a patient’s endometrium. The implementation of personalized hormone therapy is facilitated by the use of endometrial organoids. The analysis of these organoids enables clinicians to ascertain the most efficacious timing and hormone regimens, thereby enhancing the success rate of assisted reproductive technologies. The advent of multi-omics technologies has paved the way for the integration of maternal-fetal interface organoids with single-cell sequencing and spatial transcriptomics, thereby facilitating the development of personalized disease prediction models. This approach facilitates the timely identification of high-risk groups, thereby enabling the implementation of precise prevention and intervention strategies for pregnancy-related diseases.

## 5 Summary

In conclusion, maternal-fetal interface organoids have emerged as valuable models for studying early pregnancy. These three-dimensional culture systems can replicate many aspects of maternal endometrial and trophoblast biology, opening new avenues for investigating disorders such as implantation failure, preeclampsia, and infertility. However, existing organoid models have limitations: they often lack sufficient cellular diversity—omitting critical stromal, immune, or endothelial components—and depend on complex, non-standardized culture protocols, which limits their broad applicability. Future efforts should focus on incorporating multi-lineage components, improving microenvironmental fidelity, and establishing standardized protocols to enhance the translational value of these models. Currently, even if the *in vivo* environment cannot be completely replicated, different organoid systems can be customized according to specific research objectives. For example, barrier models combining trophoblast and endometrial layers can be used to study nutrient and molecule transport at the maternal-fetal interface. Likewise, immuno-competent organoids incorporating immune cells can probe the maternal-fetal immune microenvironment, and co-culture systems of trophoblast and endometrial organoids can simulate the embryo implantation process.

Looking ahead, advances in stem cell biology, multi-omics analysis, microfluidics, and computational modeling are expected to further enhance the fidelity and functionality of maternal-fetal interface organoids. With continued optimization, such models are likely to play increasingly important roles in precise disease modeling, elucidating the mechanisms of pregnancy, and drug screening. Maternal-fetal interface organoids represent not only powerful tools for uncovering the mechanisms of early pregnancy but also critical platforms for advancing personalized reproductive medicine. With ongoing development, these organoid systems hold promise for translating basic research into clinical practice, offering potential to improve the diagnosis and treatment of pregnancy complications.
